# Minimum flow decomposition guided by saturating subflows

**DOI:** 10.64898/2025.12.11.693570

**Published:** 2025-12-12

**Authors:** Ke Chen, Abhishek Talesara, Sanchal Thakkar, Mingfu Shao

**Affiliations:** 1 Department of Computer Science and Engineering, The Pennsylvania State University, PA 16803, USA; 2 Huck Institutes of the Life Sciences, The Pennsylvania State University, PA 16803, USA

## Abstract

The minimum flow decomposition problem abstracts a set of key tasks in bioinformatics, including metagenome and transcriptome assembly. These tasks, collectively known as multi-assembly, aim to reconstruct multiple genomic sequences from reads obtained from mixed samples. The reads are first organized into a directed graph (e.g., overlap graph, splice graph), where each edge has an integer weight representing the number of supporting reads. By viewing the graph as a flow network, the underlying sequences and their abundances can be extracted through decomposition into a minimum number of weighted paths. Although this problem is NP-hard, prior work has proposed an efficient heuristic that transforms the graph by identifying nontrivial equations in the flow values. However, for graphs with complex structures, many equations cannot be fully resolved by existing mechanisms, leading to suboptimal decompositions. In this study, we revisit the theoretical framework of the flow decomposition problem and extend the equation-resolving mechanisms to jointly model all equations in the graph, enabling safe merge operations that iteratively simplify the graph. Experimental results demonstrate that our new algorithm substantially improves decomposition quality over existing heuristics, achieving near-optimal solutions for complex graphs, while running several orders of magnitude faster than the ILP formulation. Source code of our algorithm is available at https://github.com/Shao-Group/catfish-LP.git.

## 1 Introduction

Minimum flow decomposition (MFD) is a fundamental problem in graph theory that asks to decompose a directed acyclic graph with edge flows into a minimum set of weighted s-t paths whose combined contributions fully explain the given flow. MFD abstracts a broad class of computational biology tasks collectively known as multi-assembly. In the general setting, sequencing reads are obtained from an unknown collection of sequences with unknown counts and are aggregated into an assembly graph; the goal is then to reconstruct those sequences and estimate their relative abundances. A prominent example is reference-based transcriptome assembly [24, 11, 23, 22, 16, 18, 15, 19, 29, 25]). For each gene, mapped RNA-seq reads are combined into a splice graph whose edge weights represent the number of reads supporting each splice junction. Decomposing this graph into s-t paths recovers the expressed RNA isoforms from the gene. Similarly, the decomposed paths can represent contigs or strain haplotypes from a microbial community in metagenomic assembly [21]; or viral strains inferred from infected hosts in the study of virus quasispecies [1].

Despite being a clean and idealized theoretical model for the above multi-assembly tasks, MFD is computationally challenging. It is known to be strongly NP-hard [26] and hard to approximate within some fixed constant factor [12]. To compute optimal solutions, Kloster et al. [14] proposed a fixed-parameter tractable (FPT) algorithm whose running time grows exponentially in the number of paths in the solution. More recently, a series of studies introduced integer linear programming (ILP) formulations for MFD [5, 9], which can handle larger instance sizes than the FPT approach and are versatile enough to incorporate extensions such as inexact flows [27, 4], safety and subpath constraints [7, 13, 28, 3], as well as graphs with cycles [6]. However, despite improvements in modeling and steady progress in commercial solvers such as Gurobi [10], the intrinsic NP-hardness of ILP limits their scalability on large practical datasets.

A complementary line of research focuses on heuristics that aim to produce practically acceptable decompositions efficiently. Among these, the greedy-width algorithm [26], which iteratively extracts the path with maximum possible flow, is widely used and often outperforms methods with stronger theoretical guarantees [12], even though it can be exponentially worse than optimal in the worst case [2]. This disconnect between theoretical guarantees and empirical performance suggests a major gap in our understanding of the structure of MFD and the behavior of its algorithms. A major step toward bridging this gap was made in catfish [20], where an efficient heuristic was introduced based on analyzing the linear algebraic structures of optimal decompositions. By identifying and resolving linear equations among edge flows, catfish substantially improves decomposition quality on both simulated and real transcriptomic graphs. The graph instances introduced in [20] have since become a standard benchmark, and even the most advanced ILP formulations still struggle to solve all of them within a reasonable amount of time.

While efficient and generally superior to greedy-width, catfish remains vulnerable to suboptimal solutions due to the presence of superficial equations—linear relations between the edge flows that do not correspond to null vectors of any minimum decomposition. Distinguishing good equations from superficial ones often requires global structural information that local flow values alone cannot capture. Our work addresses this limitation through two key insights. First, many structural dependencies among edge flows can be tested using an efficient linear programming (LP) formulation that checks whether the subflows saturating each edge are compatible with a candidate set of equations. Note that, unlike ILP, LP can be solved in polynomial time, so this filtering step incurs only modest computational overhead. Second, the feasible LP solutions obtained as a byproduct often reveal additional opportunities to further simplify a complex graph, making it possible to tackle equations that were previously unresolvable.

Building on these observations, we propose catfish-LP (Algorithm 2), an enhanced version of catfish that integrates LP-based filtering of superficial equations, safe edge-merging operations, and an LP-informed greedy extraction strategy. Through extensive experiments, we show that catfish-LP produces significantly higher-quality decompositions than previous heuristic methods, while remaining orders of magnitude faster than state-of-the-art ILP formulations.

## 2 Preliminaries

We first formally define the MFD problem. Let G=(V,E) be a directed acyclic graph with a unique source vertex s, a unique sink vertex t, and an integral flow f:E→Z. Let 𝒫 be the set of all s-t paths in G. A subset of paths P⊆𝒫 together with an integral weight function $w:P\to Z^{+}$ is called a decomposition of the flow network (G,f) if the weighted paths in P fully account for the flow in G, namely, $f(e)=\sum_{p\in P,e\in p}w(p)$ for all e∈E. The minimum flow decomposition problem asks for a decomposition (P,w) that minimizes the number of paths |P| in it. Next, we recall a few key concepts from [20] that are relevant to catfish-LP.

### 2.1 Null space of a set of paths

Assigning arbitrary indices $E=\left\{e_{1},e_{2},\ldots ,e_{\left|E\right|}\right\}$ and $𝒫=\left\{p_{1},p_{2},\ldots ,p_{\left|𝒫\right|}\right\}$ allows us to write 𝒫 as a binary matrix of size |𝒫|×|E|, where 𝒫[i,j]=1 if $p_{i}$ contains $e_{j}$ and 0 otherwise. Consequently, any subset of paths P⊆𝒫 can be viewed as a binary matrix of size |P|×|E| by keeping only the rows of 𝒫 corresponding to the paths in P. We slightly abuse notation by using 𝒫 (and P) to denote both the set of paths and the corresponding matrix; the intended meaning should be clear from context. A column vector q with |E| entries is called a null vector of a matrix P if P⋅q=0. The set of all such vectors forms the null space 𝒩(P)={q∣P⋅q=0}. For each vertex v∈V-{s,t}, its incidence vector $q_{v}\in \{0,\pm 1{\}}^{|E|\times 1}$ is defined by setting $q_{v}[i]=1$ if $e_{i}$ enters v, $q_{v}[j]=-1$ if $e_{j}$ leaves v, and $q_{v}[k]=0$ for all remaining entries. It is straightforward to verify that every $q_{v}$ is a null vector for any matrix P. We denote by ${𝒩}_{0}$ the linear space spanned by the set $\left\{q_{v}|v\in V-\{s,t\}\right\}$ and call vectors in ${𝒩}_{0}$ trivial null vectors.

### 2.2 Optimality gap

**Figure 1:**
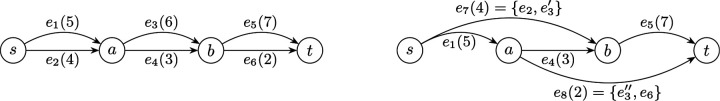
An example of reducing the optimality gap by resolving an equation. The flow value for each edge is shown in parentheses. Left: Observe that Δ=6-4+2=4. An optimal (minimum) decomposition $\left(P^{*},w^{*}\right)$ has three paths: $p_{1}^{*}=\left\{e_{1},e_{4},e_{5}\right\}$ with weight 3, $p_{2}^{*}=\left\{e_{1},e_{3},e_{6}\right\}$ with weight 2, and $p_{3}^{*}=\left\{e_{2},e_{3},e_{5}\right\}$ with weight 4. The matrix $P^{*}$ has a nontrivial null vector $(0,1,-1,0,0,1{)}^{T}$. It corresponds to an equation of flow values $f\left(e_{2}\right)+f\left(e_{6}\right)=f\left(e_{3}\right)$. Right: The equation can be resolved by splitting $e_{3}$ into $f\left(e_{3}^{\prime }\right)=4$ and $f\left(e_{3}^{\prime \prime }\right)=2$, merging $e_{2}$ with $e_{3}^{\prime }$ to get $e_{7}$, and merging $e_{3}^{\prime \prime }$ with $e_{6}$ to get $e_{8}$. In the resulting graph, Δ=5-4+2=3, so the optimality gap has been reduced from 1 to 0.

Let $\left(P^{*},w^{*}\right)$ be a minimum decomposition. An upper bound $\left|P^{*}\right|\leq \Delta =|E|-|V|+2$ is known from [26]. In [20], the authors define the optimality gap to be $\Delta -\left|P^{*}\right|$ and show that it is positive if and only if there exist null vectors outside of ${𝒩}_{0}$ (see Fig. 1 (Left) for an example):

**Proposition 1** (**Proposition 4 in** [20]) $\text{dim}\left(𝒩\left(P^{*}\right)\right)-\text{dim}\left({𝒩}_{0}\right)=\Delta -\left|P^{*}\right|$.

Since the upper bound Δ applies to any decomposition algorithm that produces independent paths, including the greedy algorithm that repeatedly selects and removes the s-t path with the largest flow value, reducing the optimality gap can potentially improve the performance of the greedy algorithm. Our goal is to apply transformations to the input flow network that reduce Δ without altering the (unknown) optimal solution. An example is shown in Fig. 1 (Right).

### 2.3 Equations of flow values

Both f and w can be represented as integral row vectors, and an equivalent formulation of (P,w) being a decomposition of (G,f) is simply f=w⋅P. Observe that for any null vector $q\in 𝒩\left(P^{*}\right)$ of a minimum decomposition $\left(P^{*},w^{*}\right)$, we have $f\cdot q=\left(w^{*}\cdot P^{*}\right)\cdot q=w^{*}\cdot \left(P^{*}\cdot q\right)=w^{*}\cdot 0=0$. Thus, each null vector corresponds to a linear equation among the flow values on edges, providing a way to identify null vectors without knowing a minimum decomposition. However, the converse does not hold. For example, in Fig. 1 (Left), the equation $f\left(e_{1}\right)+f\left(e_{6}\right)=f\left(e_{5}\right)$ corresponds to the column vector $q=(1,0,0,0,-1,1{)}^{T}$, which is not in $𝒩\left(P^{*}\right)$. Because fully resolving “good” equations—those arising from nontrivial null vectors—can reduce the optimality gap (see Fig. 1 (Right)), whereas attempting to resolve superficial equations may lead to inferior decompositions, in Section 3 we develop heuristics based on saturating subflows to identify good equations and distinguish them from superficial ones.

### 2.4 Reverse operations

**Figure 2:**
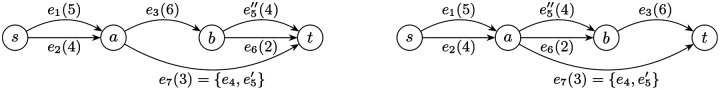
An example of fully resolving an equation with the help of a reverse operation. Left: Graph G after merging $e_{4}$ and $e_{5}^{\prime }$. Right: Graph $G^{\prime }$ obtained by reversing the subgraph between a and t.

In the example of Fig. 1, the equation $f\left(e_{2}\right)+f\left(e_{6}\right)=f\left(e_{3}\right)$ is convenient in the sense that both edges on the left-hand side are adjacent to the edge on the right-hand side, allowing the equation to be fully resolved through splitting and merging. However, many equations do not enjoy this property. For instance, it is easy to verify that the equation $f\left(e_{2}\right)+f\left(e_{4}\right)=f\left(e_{5}\right)$ in Fig. 1 (Left) also corresponds to a nontrivial null vector of the optimal solution $P^{*}$. Since $e_{4}$ and $e_{5}$ are adjacent, we may split $e_{5}$ into two edges with flow values $f\left(e_{5}^{\prime }\right)=3$ and $f\left(e_{5}^{\prime \prime }\right)=4$, and then merge $e_{4}$ with $e_{5}^{\prime }$ into a new edge $e_{7}$, producing the graph shown in Fig. 2 (Left). Note that the upper bound Δ remains 6–4+2 = 4 because the equation is only partially resolved; completing the resolution would require merging the nonadjacent edges $e_{2}$ and $e_{5}^{\prime \prime }$. To achieve this, we apply a reverse operation that “flips” a suitable subgraph, yielding a transformed graph $G^{\prime }$ in which the optimal solution is preserved. As shown in Fig. 2 (Right), $e_{2}$ and $e_{5}^{\prime \prime }$ become adjacent in $G^{\prime }$, enabling their merge and allowing the equation to be fully resolved, which in turn reduces Δ to 3.

**Figure 3:**
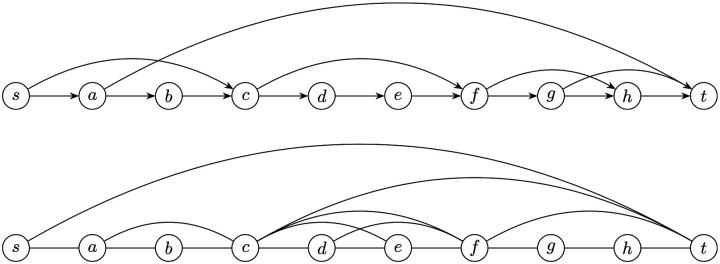
An example of closed subgraphs. Top: G=(V,E). Bottom: All closed subgraphs of G. An undirected edge between (u,v) indicates G(u,v) is a closed subgraph.

Formally, for a DAG G=(V,E) and a pair of bounding vertices (u,v), we define the subgraph G(u,v)=(V(u,v),E(u,v)) to consist of all vertices that lie on some path from u to v, together with all induced edges. Such a subgraph is said to be *closed* if every edge not in E(u,v) is either incident to u or v, or is completely disjoint from the subgraph. An example of G together with all of its closed subgraphs is shown in Fig. 3.

A closed subgraph G(u,v) can be reversed as follows: detach each edge in E(u,v) that leaves u and reattach it to v; detach each edge in E(u,v) that enters v and reattach it to u; and then flip the direction of every edges in E(u,v). Let $G^{\prime }$ be the resulting graph, the following proposition shows that the reverse operation preserves all possible decompositions:

**Proposition 2** (**Proposition 5 in** [20]) *There exists a one*-*to*-*one correspondence between all decompositions of* (G,f) *and all decompositions of* $\left(G^{\prime },f\right)$.

Moreover, all closed subgraphs of G can be computed in $O\left(\left|V{|}^{2}\cdot |E\right|\right)$ time, and a sequence of reverse operations that makes any two given edges adjacent (when possible) can be computed within the same time bound. This is the main mechanism we employ to resolve equations involving nonadjacent edges.

## 3 Method

Given the insight that resolving equations can reduce the optimality gap and thus improve the quality of the greedy decomposition, together with the machinery developed for using reverse operations to perform such resolutions, we arrive at the following natural heuristic algorithm.


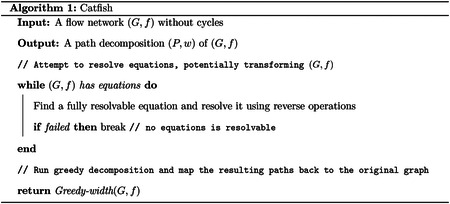


Recall that the optimality gap decreases only when we resolve good equations that arise from nontrivial null vectors of an unknown minimum decomposition. However, not all equations are good. Prior work [20] shows that, under certain uniformity assumptions, the probability of encountering a superficial equation on edge flow values (one that does not correspond to a nontrivial null vector) is low. Consequently, when the graph structure is simple enough such that most equations are good, Algorithm 1 can produce optimal or near-optimal decompositions. In more complex settings, on the other hand, superficial equations are more likely to be selected, often leading to suboptimal solutions. To mitigate this issue, we introduce the following linear programming (LP) formulation, which tests a necessary condition for equations to be good.

### 3.1 The base model

Let m=|E| be the number of edges in G. The base model introduces $m^{2}$ continuous variables: for each edge e∈E, we define a set of m variables $x_{e}(\cdot )$, one for each edge in the graph. Intuitively, $x_{e}(\cdot )$ is intended to represent a valid s-t-subflow in G that saturates edge e; equivalently, it encodes the collective flow of all paths in a decomposition that traverse e. We enforce this interpretation through the following constraints:

**Constraint 1.** $x_{e}(e)=f(e)$ for all e∈E: the subflow associated with $x_{e}(\cdot )$ saturates edge e.
**Constraint 2.** $\sum_{(s,v)\in E}x_{e}((s,v))=x_{e}(e)$: the subflow defined by $x_{e}(\cdot )$ must all pass through the edge e.
**Constraint 3.** $0\leq x_{e}(a)\leq f(a)$ for all e,a∈E: each edge in each subflow carries a valid amount of flow.
**Constraint 4.** $\sum_{(u,v)\in E}x_{e}((u,v))=\sum_{(v,w)\in E}x_{e}((v,w))$ for all e∈E and all v∈V-{s,t}: each subflow satisfies flow conservation at every intermediate vertex.

Note that we do not impose constraints of the form $\sum_{e\in E}x_{e}(a)\leq f(a)$, since subflows may overlap. For example, if a path in $P^{*}$ saturates two edges $e_{1}$ and $e_{2}$, then the subflows defined by $x_{e_{1}}(\cdot )$ and $x_{e_{2}}(\cdot )$ should coincide along the entire path; in particular, $x_{e_{1}}\left(e_{1}\right)+x_{e_{2}}\left(e_{1}\right)=2f\left(e_{1}\right)>f\left(e_{1}\right)$. At the same time, subflows must not be entirely independent. To encode this dependency, we further impose the following symmetry constraint:

**Constraint 5.** $x_{e}(a)=x_{a}(e)$ for all edges e,a∈E: both variables represent the total amount of flow that passes through both edges e and a.

### 3.2 Equation constraints

We consider only equations on edge flow values with coefficients in {0,±1}. Such an equation can be specified by two subsets $E_{L},E_{R}\subseteq E$: writing $f\left(E_{1}\right)=\sum_{e\in E_{1}}f(e)$ for any $E_{1}\subseteq E$, the equation takes the form $f\left(E_{L}\right)=f\left(E_{R}\right)$. Since linear combinations of equations are also equations, we restrict attention to indivisible equations—those that admit no strict subsets $E_{L}^{\prime }\subset E_{L}$ and $E_{R}^{\prime }\subset E_{R}$ with $f\left(E_{L}^{\prime }\right)=f\left(E_{R}^{\prime }\right)$—because they are smaller in size and already encode all relevant information. Note that being indivisible implies that $E_{L}$ and $E_{R}$ are disjoint. If an indivisible equation $f\left(E_{L}\right)=f\left(E_{R}\right)$ indeed arises from a nontrivial null vector of some minimum decomposition $P^{*}$, then it captures the structural dependency that any path in $P^{*}$ using an edge in $E_{L}$ must also use some edges in $E_{R}$, and vice versa. Moreover, the subflow saturating an edge in $E_{L}$ should not use any other edges of $E_{L}$ (same for $E_{R}$). To model these relationships, we add the following 2m constraints for an equation:

**Constraint 6.** $\sum_{e\in E_{L}}\;x_{e}(a)=\sum_{e\in E_{R}}x_{e}(a)$ for all a∈E: for each edge, the total subflow through edges in $E_{L}$ must equal that through edges in $E_{R}$.
**Constraint 7.** $\sum_{e\in E_{L}}x_{e}(a)\leq f(a)$ for all a∈E: the combined subflow contributed by edges in $E_{L}$ (and therefore $E_{R}$) may not exceed the total flow on each edge.

This LP formulation checks a necessary condition for the equation $f\left(E_{L}\right)=f\left(E_{R}\right)$ to correspond to a nontrivial null vector of a minimum decomposition $P^{*}$: in the equation is valid for $P^{*}$, then the induced subflows saturating each edge (obtained by aggregating all paths in $P^{*}$ that use the edge) satisfy all the above constraints, and hence the LP model is feasible. Taking the contrapositive, an infeasible LP instance certifies that the equation cannot arise from a nontrivial null vector of any minimum decomposition, and thus the equation should be discarded.

Better yet, this formulation also allows us to test whether a set of equations is compatible (since all good equations arising from nontrivial null vectors of $P^{*}$ must be mutually compatible). Starting from the base model, we can add equation constraints sequentially and check feasibility at each step. If the LP becomes infeasible after adding a particular equation, that equation (and its associated constraints) is removed. This procedure further filters out superficial equations and thereby improves the quality of the final decomposition. It is worthnoting that the LP model could be strengthened to check for more conservative conditions, for example, by adapting the ILP formulation of [5]. However, our experiments indicate that the proposed LP model is orders of magnitude more efficient, and its filtering quality is already sufficient for practical use.

### 3.3 Transform G with a feasible LP

The LP-based verification can be directly integrated into Algorithm 1. The primary remaining bottleneck is that not all good equations can be successfully resolved through reverse operations, especially when the graph structure is complex. A feasible LP model after filtering out superficial equations, provides valuable structural information that can be used to simplify the graph. When no equation can be fully resolved, we apply the following two heuristics:

1. Recall that a feasible LP solution provides, for each edge e∈E, a valid subflow $x_{e}(\cdot )$ that saturates e. If, in this subflow, an edge $e^{\prime }$ satisfies $x_{e}\left(e^{\prime }\right)=f(e)$, then there exists a valid decomposition (consistent with all remaining equations) in which every unit of flow through e also uses $e^{\prime }$. Moreover, if e and $e^{\prime }$ are adjacent, they can be conveniently merged while preserving the decomposition. However, this merge may be only supported by some feasible decompositions discovered by the LP and is not guaranteed to be correct for an optimal one. To err on the safe side, we again use LP to determine whether e and $e^{\prime }$ must be merged in all decompositions consistent with the current equation set. This can be done by adding a temporary constraint $x_{e}\left(e^{\prime }\right)<f(e)-\epsilon$ and test for feasibility. If the LP becomes infeasible, the merge is deemed safe, and we contract e and $e^{\prime }$ to simplify the graph. Such merges can have meaningful downstream effects, for example, enabling the resolution of equations that are otherwise unresolvable.
2. If no further safe simplifications are possible, the algorithm exits the while-loop and proceeds to greedy decomposition. Empirically, we observe that greedy decomposition almost always yields inferior solutions when unresolvable equations remain. In such cases, the feasible LP solution itself may lead to a better outcome. We examine the LP solution to identify any saturating subflow $x_{e}(\cdot )$ that is actually a simple path through e. Among all such paths, we extract the one carrying the maximum flow f(e), remove it from the graph, and add it to the final decomposition. Unlike the previous heuristic, this operation is not guaranteed to be safe as the extracted path may not appear in all compatible decompositions. Instead, it serves as an “informed greedy” step: selecting the heaviest path that is consistent with all remaining equations, rather than the heaviest path in the graph overall.

### 3.4 The complete algorithm


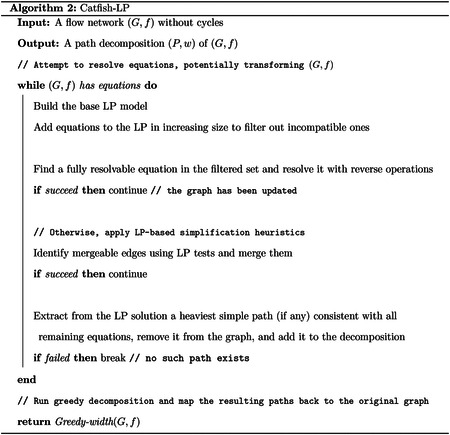


## 4 Experimental Results

We evaluate four algorithms for the MFD problem: the greedy-width and catfish algorithms as implemented in [20], the optimized ILP formulation from [9], and our enhanced catfish-LP. All experiments are conducted on an Ubuntu server equipped with an Intel(R) Xeon(R) Gold 6148 CPU @ 2.40GHz and 566 GB RAM. Both ILP and catfish-LP rely on the same Gurobi installation; ILP uses the Python API and catfish-LP uses the C++ interface. Each method is limited to at most five cores per instance, and total CPU time is recorded using time(1) -v. For each instance, ILP requires a valid initial decomposition to enable its safety optimizations, for which we supply the greedy-width solution. Since the running time of greedy-width is negligible compared to ILP, its execution time is not included in ILP’s total time. A wall-clock time limit of 30 minutes is imposed for each instance.

### 4.1 Datasets

We evaluate all methods on four datasets of perfect splice graphs generated by [20]. The first dataset is derived from human RNA-seq samples quantified using Salmon [17]. For each gene in each sample, perfect splice graphs are constructed by superimposing all expressed transcripts with their estimated abundances. We refer to this dataset as Salmon.

The remaining three datasets are generated via simulation using the Flux-Simulator [8] on three well-annotated species: human, mouse, and zebrafish. We refer to these datasets by the corresponding species name.

In addition, we generated 20 simulated graphs for each of 72 configurations, varying parameters such as the number of vertices, the number of ground-truth paths, and the maximum length of each path. This simulation dataset allows us to systematically evaluate algorithm performance across a range of graph complexities and difficulty levels. We refer to this dataset as Simulation.

### 4.2 Results on perfect splice graphs

Table 1 summarizes the decomposition quality of each method on the four datasets. Because the input graphs are constructed from ground-truth paths, we use the total number of such paths, $\left|P^{†}\right|$, as a baseline for evaluating all algorithms. Note that $\left|P^{†}\right|$ does not necessarily correspond to the minimum possible decomposition; indeed, both Catfish-LP and ILP frequently produce even smaller solutions. However, the ground-truth paths are biologically meaningful: in transcript assembly, pursuing a strictly minimum decomposition is somewhat arbitrary, whereas recovering the true underlying isoforms is a more relevant objective (though difficult to assess on real data where ground truth is unavailable). For this reason, we also report the recall of each algorithm, defined as the fraction of ground-truth paths that are correctly recovered. A decomposed path is considered correct if it matches a ground-truth path exactly in both its node sequence and its weight.

Observe that catfish-LP outperforms both greedy-width and catfish in producing smaller decompositions, often approaching the exact solutions obtained by ILP. At the same time, catfish-LP consistently achieves the highest recall across all datasets, suggesting that the saturating subflows it relies on successfully capture biologically meaningful structural information within splice graphs.

Table 2 reports the CPU time of all methods on the four datasets. As expected, catfish-LP is slower than catfish due to the additional LP-based filtering, which is the key contributor to its improved decomposition quality. Nonetheless, because LP is polynomial-time solvable, catfish-LP remains orders of magnitude faster than ILP. Among the four methods, only ILP exceeds the 30-minute time limit on certain instances, suggesting that its true time to completion would, in many cases, be even larger.

### 4.3 Results on simulated graphs

As reported in [20], and as reflected in the second and third columns of Table 1, most perfect splice graphs in the above datasets are relatively simple: the vast majority admit ground-truth decompositions of at most ten paths. To better assess algorithmic performance on more challenging instances, we generated additional synthetic graphs with 20 or 30 vertices, between 10 and 30 ground-truth paths, and maximum path lengths ranging from 10 to 23. Increasing the number and length of ground-truth paths on a fixed vertex set naturally creates more entangled graph structures, making accurate decomposition substantially harder.

**Figure 4:**
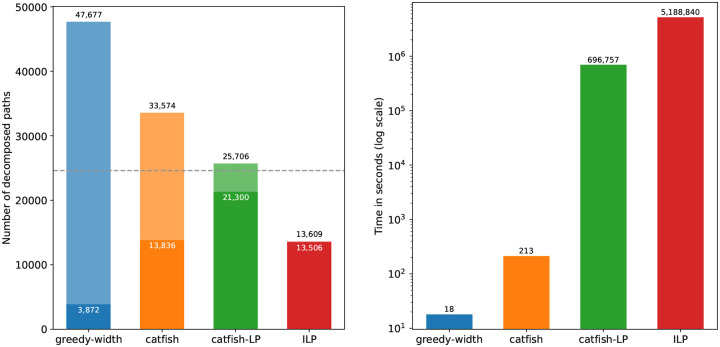
Decomposition quality and runtime on the simulated dataset. In the left subfigure, the dashed horizontal line marks the total number of ground-truth paths across all instances $\left(\left|P^{†}\right|=24,600\right)$. For each method, the full bar represents the total number of paths produced, while the shaded portion denotes the number of decomposed paths that exactly match a ground-truth path. The ILP bar is heavily skewed because ILP failed to decompose 498 graph instances (35%) within the time limit.

Fig. 4 summarizes both decomposition quality and running time on this simulated dataset. Observe that catfish-LP consistently produces smaller decompositions and recovers more ground-truth paths than all other heuristic methods. ILP, by contrast, fails to finish within the time limit on a large fraction of the more complex graphs—precisely the instances that also challenge the heuristic methods—making its results less directly comparable in this experiment. This is also evident from its runtime profile: over 85% of ILP’s total reported CPU time arises from timeout instances. On the remaining simpler graphs where ILP finishes, the total runtime is 706,840 seconds, during which all heuristic algorithms complete the entire dataset.

## 5 Summary and discussion

This work introduced catfish-LP, a new algorithm for the minimum flow decomposition problem that augments the state-of-the-art heuristic framework, catfish, with a lightweight yet highly informative use of linear programming. By leveraging LP both as a filter for identifying structurally consistent equations and as a guide for graph simplification via saturating subflows, catfish-LP addresses a central challenge in the theoretically motivated equation-resolving-based strategy: determining which equations are valid for an unknown optimal decomposition and how to exploit them when direct resolution fails. Unlike ILP-based approaches, which offer exact solutions but scale poorly, our LP-based design remains computationally efficient while capturing much of the structural insight needed for high-quality decompositions.

Across a broad collection of datasets, including splice graphs derived from real RNA-seq data and a controlled suite of simulated graphs with diverse levels of complexity, catfish-LP consistently produces decompositions that are nearly as small as those obtained by ILP while achieving substantially higher recall, outperforming classical heuristics such as greedy-width and the original catfish. These improvements validate the central premise of the method: feasible LP solutions expose structurally meaningful saturating subflows that can validate good equations, guide safe graph transformations, and enable informed greedy extractions in situations where purely combinatorial heuristics frequently stuck or make suboptimal choices.

Our experiments further show that catfish-LP provides a compelling balance between accuracy and scalability. Although slower than the purely combinatorial catfish algorithm due to the necessary LP invocations, it remains orders of magnitude faster than ILP and scales effectively to large and entangled graphs where ILP routinely hits time limits. Moreover, the LP formulation introduced here provide a flexible foundation that can be adapted to other MFD variants with meaningful bioinformatics applications.

Overall, catfish-LP demonstrates that incorporating LP into the combinatorial structure of MFD yields both practical benefits and conceptual insights. Practically, it produces smaller and more accurate decompositions within realistic computational budgets. Conceptually, it highlights how LP-feasible subflows can encode structural information about the decomposition space, suggesting new directions for hybrid optimization–combinatorial approaches for MFD and related flow-based problems. Future directions include exploring strengthened LP formulations, integrating domain-specific constraints, and developing probabilistic interpretations of LP-guided decompositions for transcriptome assembly and beyond.

## Figures and Tables

**Table 1:** Decomposition results on perfect splice graphs. The column $\left|P^{†}\right|$ shows the total number of ground-truth paths (RNA isoforms) in each dataset. Recall is computed as the percentage of correctly decomposed paths.

Dataset	Graphs	$\left|P^{†}\right|$	$|P|/\left|P^{†}\right|$				Recall%			
			greedy-width	catfish	catfish-LP	ILP	greedy-width	catfish	catfish-LP	ILP
Salmon	13,300,893	32,889,145	1.0404	1.0026	1.0005	0.9983	87.37	96.11	96.58	96.51
human	1,169,083	2,139,559	1.0062	1.0002	0.9999	0.9996	95.99	97.75	97.80	97.69
mouse	1,316,058	2,142,500	1.0050	1.0000	0.9996	0.9993	96.20	97.72	97.81	97.61
zebrafish	1,549,373	2,140,200	1.0010	0.9998	0.9998	0.9998	98.45	98.90	98.90	98.80

**Table 2:** Running time on perfect splice graphs. The ILP timeout column shows the number of instances/total number of ground truth paths in those instances, for which ILP failed to produce a decomposition within the 30 minutes time limit.

Dataset	CPU time (seconds)				
	greedy-width	catfish	catfish-LP	ILP	ILP timeout
Salmon	2,713	2,945	158,862	24,182,561	2,106/43,476
human	174	159	7,772	179,445	12/169
mouse	178	152	8,820	216,275	15/233
zebrafish	195	151	9,752	13,421	1/16
